# Author’s Response to Peer Review Reports on “Investigating the Variable Component of the Systematic Error, a Neglected Error Parameter: Theoretical Reevaluation Study”

**DOI:** 10.2196/88981

**Published:** 2026-02-27

**Authors:** Atilla Barna Vandra

**Affiliations:** 1 Spitalul Clinic Judetean de Urgenta Brasov, Str. Berzei 2 Bl. B. ap 20, Brasov, 500276, Romania, 40 722264666

**Keywords:** repeatability condition, reproducibility within laboratory condition, measurement, systematic error, clinical laboratory, quality control, bias, QC, statistical, statistics, mathematics, computer simulation, standard deviation

*This is the author’s response to peer-review reports on “Investigating the Variable Component of the Systematic Error, a Neglected Error Parameter: Theoretical Reevaluation Study.”*

## Round 1 Review

### Reviewer C [1]

*1. The handling of constant or intermittent bias has been a challenge for more than 200 years, especially since Gauss and Laplace’s work in the early 19th century. The author of this paper [2] refers to Eisenhart’s excellent 1963 paper, which is appropriate but not as the origin of the variable component of the systematic error (VCSE). Shewhart’s 1923 and 1939 books [3,4] also address this matter.*

**Response:** Thanks for the information about the first constant or variable bias debates. I have changed the text referring to C Eisenhart as the first author, who mentioned VCSE, referring to Shewhart, mentioning him in the reference list.

*2. Bias, including VCSE, is also a major point of contention in the International Bureau of Weights and Measures/International Organization for Standardization work on the Guide to the Expression of Uncertainty in Measurement (GUM) and its revision. The complexity of the issues is illustrated, for example, in a book by Krystek [5]. The current manuscript illustrates the opinions of its author but fails to illustrate the background of the immense scientific literature and debates that have already dealt with the matter*.

**Response:** I have found in JCGM (Joint Committee for Guides in Metrology) GUM-6:2020 paragraph 10.6 a discussion about drift effects and compared it with JCGM 100:2008 GUM 3.2.4 recommendations, which hide the assumption of a constant bias. Neither a correction (B.2.23) nor a correction factor (B.2.24) can eliminate a function (a time-variable bias). Please take a look at Principle 3 in the new version. I have downloaded and read the study by Krystek and refer to it in the new version.

*3. The author needs to clarify whether he adheres to the error or uncertainty paradigms in measurement uncertainty/error questions. The current manuscript represents a mixture of both*.

**Response:** I discuss in the new version that the total measurement error (TE) and uncertainty of measurement (MU) paradigms are, in essence, complementary, not contradictory. (This was also mentioned in the old version; I tried to be more explicit). TE and MU are linked to two different points of view. The TE approach cannot challenge the MU in calculating the MU, nor can the MU approach challenge TE in short-term decisions, as in internal quality control decisions. More than that, the two approaches have common parts, being based on the same oversimplified error model, and corrections influence both theories.

*4. The “thought-provoking and even shocking” fact that the Westgard rules and power calculations are based on repeatability uncertainty and not on reproducibility uncertainty is well-known by metrologists in clinical chemistry. Unfortunately, mentioning this fact commonly hurts the sentiments of a majority in our field, and many of us avoid harping on it. A prerequisite for appropriately using Westgard rules and variants is that changing goal mean values are used as calibrators, and reagent lots change over time*.

**Response:** Quoting his text: “The ‘thought-provoking and even shocking’ fact that the Westgard rules and power calculations are based on repeatability uncertainty and not on reproducibility uncertainty is well-known by metrologists in clinical chemistry.” I have reformulated, eliminating the label, referring only to the recommendations of Westgard et al [6] to design the Levey-Jennings graphs based on the SD calculated from long-term control data (“at least 20 measurements over at least two weeks or ten working days, and preferably over at least four weeks or 20 working days”). I did mention the CLSI C24-Ed4 2016 recommendations: “From a clinical point of view, repeatability is rarely of interest. Generally, within-laboratory precision estimates are clinically more relevant because they reflect variability over time intervals somewhat more representative of intervals between repeat measurements for a patient being monitored for chronic disease.” Also: “The Sigma metric may be calculated using either repeatability or within-laboratory imprecision as the estimate of the SD. However, for the most useful estimates of the Sigma metric, the within-laboratory SD is the best choice.” I also mentioned the paper of Westgard and Groth [7], in which they accept that the power function graphs are designed with s_r_ (SD measured in constant, repeatability conditions). Seemingly, they knew the contradiction between the Levey-Jennings graphs and the power function graphs but continued to suggest creating the Levey-Jennings graphs with sRW (SD measured in variable, reproducibility within laboratory conditions).

*5. The traceability hierarchies used when producing reference materials and calibrators are usually claimed to explain the variations experiences (eg, during lot-number changes). The author apparently does not accept this explananation of the main cause of lot-number shifts/bias, and he needs to explain why his mathematical/statistical theory should be accepted instead*.

**Response:** I did not state that I do not accept that the nominal value errors (with the words of reviewer C: “The traceability hierarchies used when producing reference materials and calibrators” are not sources of “the variations experiences [eg, during lot-number changes]”) (with other words of the VCSE(t) [variable component of systematic error at the moment t]). More than that, I have sustained that shifts caused by the calibrations are one of the causes of the VCSE(t). Lot number changes are only one of the causes of shifts after calibrations. The revised manuscript also details other causes, such as the measurement error during calibration and the reconstitution errors linked to the reference material bottles. My theory does not contradict the importance of the nominal value errors; more than that, it completes it. I have stated: In the time frames between human interventions, the bias variations are predictable, hidden behind the noise of the RE (random error component). A lot of change is a human intervention. This is one of the causes of the bias variation. Both lot number changes and control bottle changes (reconstitution error) cause shifts in bias.

*6*. *In a crucial part of his manuscript, the author claims that “While RE changes unpredictably from measurement to measurement, VCSE(t) remains quasi-constant in a given day, influencing all measurement results obtained in that day systematically. But in long-term experiments, VCSE(t) becomes a cyclical time-variable function, which repeats the same values after unequal periods. (A period may last even one month).” The author presents Cobas 6000 analyzer data in support of his thesis. However, data from a variety of measuring systems, lot changes, and measurands are needed before this theory of a cyclical phenomenon is chosen instead of a theory of random components*.

**Response:** I have detailed the idea of cyclical variation in the revised version. I hope I did it more explicitly. The reagent property changes are unidirectional, causing drifts. The bias increase (in absolute values) cannot continue endlessly because shifts caused by human interventions correct it (reagent changes, calibrations). The consequence is a sawtooth-like graph, a cyclical variation as in Figure 3. I have substituted Figure 3 (preprint version) with two others (one real-life), explaining that only in the case of significant drifts can the sawtooth-like character be observed behind the noise of the random errors. Without a known cause, the causes of SD increase in time were labeled “random.” However, we cannot identify any causes of variable RE (the sRW variations are caused not by RE but by the VCSE(t)). I have underlined that the presented phenomenology was observed on all analyzers I have worked with. Also, I highlighted that the given examples can be visually observed only if they are significant (usually, the phenomena are hidden behind the RE). Because the real cause was unknown, the myth of random bias variations was born.

### Anonymous [8]

*1. The study does not provide empirical confirmation of suggested approaches using real-world data, although it mentions computer simulations and experimental verification. The suggested methodologies’ efficacy and dependability are yet unknown in the absence of empirical validation*.

**Response:** Thanks for the idea. I did not want to introduce more graphs because of the length of the study, but in the revised version, I shall do it.

*2. Linear drifts in daily means across time are assumed in the study. Numerous factors, such as the environment, instrument calibration, and reagent stability, can affect real-world drift patterns and lead to nonlinear trends in daily means over time. The study might have simplified the complicated nature of drift processes by assuming linearity, which could result in estimates of mean values and error components that are not true*.

**Response:** Thanks for the idea. In the revised version, I shall explain why environmental changes have an insignificant influence on thermostated reactions in an automatized laboratory with air conditioning. “Human (operator) errors” and “laboratory errors” are redundant in the error list because they always act via instrumental and noninstrumental errors. Bias variations are always specific to the reaction; therefore, they may have only noninstrumental causes (reagent stability and calibration curve changes). The quasi-linear drifts are caused only by the reagent property changes. Random variations in reagent properties would contradict the laws of chemistry. Calibrations cause shifts, which have known moments (human interventions) but to a random extent. The “linear drifts in daily means” happen in the intervals of the human interventions (calibrations, regent changes, control bottle changes). Across these, we cannot apply the quality control rules. This is also the recommendation of Westgard. However, there are unexpected shifts (eg, caused by carry-over phenomena), and the quality control must be able to detect them.

*3. The assumption that information from internal quality control sources alone can be used to accurately calculate VCSE(t) is inaccurate. Even though internal quality control data offer insightful information on short-term variability, they might not include all sources of variation, particularly those pertaining to outside variables like environmental shifts, instrument performance, or operator technique. Ignoring these outside influences could result in an inaccurate or understated VCSE(t), which would compromise the validity of the suggested quality control techniques*.

**Response:** Thanks for the observation. In the revised version, I shall provide details (see the former answer to observation 2). The anonymous reviewer is right; VCSE(t), by definition, is variable, but so is sVCSE. VCSE(t), the value of the VCSE in the moment t, can be determined accurately within the limits of the statistical methods, but it only has 24-hour validity. I agree that its determination has a high cost/effectiveness ratio. Its approximate evaluation in the internal quality control is a better choice. sVCSE depends on the time frame. Therefore, its determination has acceptable accuracy only from yearly data. The method was described. Because sVCSE depends on the time frame, its value cannot be used only in the same time frame in which it was determined.

As will be detailed more in the revised version, the importance of the VCSE(t) and sVCSE is not their value but the knowledge about their existence. We can avoid redundant use by highlighting these error components in equations. In calculations, they are summed with other error components (ie, are “hidden” in Br(t) or sRW); therefore, their absolute values are not important. However, it is essential to avoid the use of Br(t) and sRW in the same equations (eg, TE = Br(t) + z*sRW).

*4. Although there is a suggestion in the Conclusions section that the present quality control paradigm needs to be revised, there is no concrete plan or set of recommendations based on statistical or mathematical concepts*.

**Response:** Thanks. I will make recommendations based on the concepts presented in the study to improve the quality control. The next step is to present a new quality control system.

*5. The paper lacks a Discussion section, which could have allowed the author to interpret and contextualize the study’s findings. Additionally, it could have provided an opportunity to compare the study’s findings with previous studies, discuss their implications, and address potential sources of error or bias*.

**Response:** The revised version will have a more detailed Discussion section, and the paper will be reorganized. The implications were discussed: separating the bias components prevents the VCSE(t) redundancy in equations and suggested corrected equations. The revised version will be more explicit in presenting the bias sources: the reagent instability and the calibration graph errors. I also included a Comparison with the Literature section.

## Round 2 Review

### Reviewer C

*In the first round of reviews, I asked for “(1) a well-structured manuscript based on (2) extensive knowledge of the state of the art in calculating measurement uncertainty and (3) well-written English text.”*

*The revised version of the manuscript has improved the English text but needs to improve in the two other aspects.*

*I agree that the paper’s subject is essential. The author is well-versed in mathematical statistics and has practical experience in laboratory quality control. However, the manuscript lacks in:*

1. *Counting in metrological aspects*
2. *Using a conventional manuscript structure*
3. *Showing sufficient real laboratory results and the consequence of using the proposed paradigm on real laboratory results*

**Response:** I shall begin with point 2.

I am quoting Reviewer C: “I asked for “(1) a well-structured manuscript…” “The manuscript lacks a conventional manuscript structure.”

I have rewritten the whole manuscript, trying to respect the required structure. I thought that both the expressions and the content are important. I have given slightly different names, with the same sense. The titles of the main sections were Introduction (introduction), Methods (materials and methods), Results (experimental data and computer simulation), Discussion (discussion), Conclusions (conclusions).

Each section has subsections. I divided them into subsections to make it easier to trace the experimental data.

In the Methods section, three experiments were described, showing three different phenomena. In the Results section, each has a different subsection. The Discussion section was divided into five subsections, each discussing various aspects.

I have renamed the main sections, and I numbered the subsections. I hope it is OK now.

I am quoting Reviewer C: (I asked for) “extensive knowledge of the state of the art in calculating measurement uncertainty.”

The anonymous reviewer, in the first review round, asked me to adhere to one of the paradigms: TE or UM. I answered him/her that I did not want to because the two paradigms have different areas of use and are linked to two different points of view: short- and long-term. Neither can substitute the other in its area of applicability. However, this study focuses on internal quality control decisions, which are short-term decisions, and therefore, the study focuses on TE. However, changing the error model has consequences for both paradigms because both are built on the same error model. This theoretical study focuses on equations and mathematics, not applicability. Briefly: bias is variable; it is a time-variable function, and we must make a distinction between bias types. Otherwise, there is the risk of redundant use. Bias variability has two sources; both are noninstrumental: the reagent instability and the calibration errors. s_r_ is the estimator of the true RE, and sRW is erroneously considered the measure of the RE because it is the measure of all variable components (RE + VCSE). There is nothing about the uncertainty of measurement. I have mentioned several times that further studies are necessary to analyze the applicability of the new error model. These analyses neither fit the study’s task nor its word count limits. The aim was to be the foundation stone for a new quality control system. I have mentioned the guiding principles, but this study is not about presenting and proving the applicability of the new system. I cannot publish a new quality control system without the new error model. I have been waiting two years to publish it, but I cannot until the error model is published. To detail the hidden false assumptions of the Westgard-rules–based quality control system, I need the help of the new error model. To detail all principles, too. I need space to present a new rule system based on a modified Levey-Jennings graph and sustain it with computer simulation data and probability calculations (Westgard et al also did not present their own quality control system with real-life data; it was only based on computer simulations). However, I have proofs based on real-life data. These cannot be presented before the former analysis and descriptions are presented. This study is a foundation stone, not a new method, and neither of these are based on UM.

I suppose, but I am not sure, that Reviewer C wants an analysis of the error model based on GUM. The UM starts with correcting the discovered biases in the first step using corrections and correction factors. But a constant cannot fix a variable!! A single bias value does not give information about the constant or proportional character of the bias. A smaller bias than its uncertainty cannot be corrected. Bias is variable because of its properties, and this variability is significant in the clinical laboratory. The external quality assessment (EQA) results are obtained after a considerable delay while calibrations and reagent changes are done. A correction applied after such a delay is risky! The UM strategy may be efficient in lifeless domains but not in the conditions of the variability of biases in the clinical laboratory. Because of the delay, we do not correct the measured bias based on the last EQA, but a significantly different one! Such corrections assume a constant bias, which is a false assumption. The analysis of such contradictions neither fits the study’s aims nor its limits, mainly because Reviewer C asks for proof of real-life data. Therefore, a presentation of the UM literature, in my opinion, is unnecessary.

The error model also has consequences for UM equations. To prove that the UM equations are based on “the bias” definitional uncertainty is not about the error model, but rather, it is a review of the UM. It is not the task of this study, which is about an error model. It is not the same as correcting a bias value or a mean bias. The uncertainty of a value and a mean is different, too. The UM equations include neither the uncertainty caused by the reconstitution error of the reference material, which is usually bigger than the uncertainty of its nominal value, nor the uncertainty of the sRW (which may be double in the next month). This is not a review paper. The literature about conditions not respected for a correct EQA does not fit in a single room. The most used methods (because of the unavailability of commutable certified materials and economic reasons) are using peer group means as surrogate reference values. The formula for calculating the nominal value uncertainty and the target value is invalid in these groups. Therefore, there are two different uCref values, one declared (erroneously calculated) and the other real. The latter is included in RMSbias. RMSbias + uCref redundantly uses the uCref term (one is bottom-up, the other is an up-down parameter, which cannot be mixed without redundancy…let me continue?)

I have included only a brief (two pages) presentation of the upper analysis. However, it is a cuckoo’s egg. Neither fits in the task of a theoretical study about an error model nor real-life data proofs that the corrections based on the last EQA increase the variability of biases (instead of reducing them), contributing to the increase of total uncertainty. (I have made some simulations based on real-life data (based on four-year EQA data), and this was the conclusion: only mean biases can be used in corrections, single EQA data cannot). The presentation of these does not fit in the limits of this study.

My question remains: what do I need to prove with real-life data? That bias is variable? Even if it is accepted that it is variable, I need to prove with real-life data that we must make a difference between different biases. That the variable bias does not fit into the classical error model? That calibrations and reagent property changes cause bias variations? All these were described in the literature in mosaic pieces. A quality control system based on s_r_ and the avoidance of alarms in the case of incorrigible biases USING DIFFERENT RULES, not the actual Westgard rules, and the fact that correctly applied Westgard rules are an unfunctional quality control system, does not fit into the limits of this study.

I am quoting Prof A Marusteri: “If this is true, nothing we thought certain points in quality control are unquestionable anymore.” With such a radical change, several phenomena change in quality control. Each needs proof. However, so many things cannot be presented in a study. Therefore, I have mentioned several times that something “needs a separate study.”

*3. “However, the manuscript lacks in “Counting in metrological aspects.”*

**Response:** I do not understand this critique; I do not understand what the statement refers to.

## Abbreviations

EQA: external quality assessment
GUM: Guide to the Expression of Uncertainty in Measurement
JCGM: Joint Committee for Guides in Metrology
MU: uncertainty of measurement
RE: random error component
s_r_: SD measured in constant, repeatability conditions
sRW: SD measured in variable, reproducibility within laboratory conditions
TE: total measurement error
VCSE: variable component of the systematic error
VCSE(t): variable component of systematic error at the moment t
